# Supplementary material for: OpenMM 7: Rapid development of high performance algorithms for molecular dynamics
Source: PLoS Comput Biol. 2017 Jul 26;13(7):e1005659. doi: 10.1371/journal.pcbi.1005659 (PMC5549999; doi:10.1371/journal.pcbi.1005659)
Supplement: S1 Source Code — (ZIP) [file pcbi.1005659.s002.zip › OpenMM-7.0.1-Source/docs/api-python/generated/simtk.openmm.openmm.CMMotionRemover.html]

CMMotionRemover — OpenMM 7.0.1.dev-5e86c4f documentation


# CMMotionRemover¶

*class* `simtk.openmm.openmm.``CMMotionRemover`(*\*args*)¶
:   This class prevents the center of mass of a System from drifting. At each time step, it calculates the center of mass momentum, then adjusts the individual particle velocities to make it zero.

    `__init__`(*self*, *frequency=1*) → CMMotionRemover¶
    :   \_\_init\_\_(self) -> CMMotionRemover
        \_\_init\_\_(self, other) -> CMMotionRemover

        Create a CMMotionRemover.

    Methods

    |  |  |
    | --- | --- |
    | `__init__`((self[, frequency]) | \_\_init\_\_(self) -> CMMotionRemover |
    | `getForceGroup`((self) -> int) | Get the force group this Force belongs to. |
    | `getFrequency`((self) -> int) | Get the frequency (in time steps) at which center of mass motion should be removed |
    | `setForceGroup`(self, group) | Set the force group this Force belongs to. |
    | `setFrequency`(self, freq) | Set the frequency (in time steps) at which center of mass motion should be removed |
    | `usesPeriodicBoundaryConditions`((self) -> bool) | Returns whether or not this force makes use of periodic boundary conditions. |

    `getFrequency`(*self*) → int¶
    :   Get the frequency (in time steps) at which center of mass motion should be removed

    `setFrequency`(*self*, *freq*)¶
    :   Set the frequency (in time steps) at which center of mass motion should be removed

    `usesPeriodicBoundaryConditions`(*self*) → bool¶
    :   Returns whether or not this force makes use of periodic boundary conditions.

        |  |  |
        | --- | --- |
        | Returns: | true if force uses PBC and false otherwise |
        | Return type: | bool |

    `__delattr__`¶
    :   x.\_\_delattr\_\_(‘name’) <==> del x.name

    `__format__`()¶
    :   default object formatter

    `__getattribute__`¶
    :   x.\_\_getattribute\_\_(‘name’) <==> x.name

    `__hash__`¶

    `__reduce__`()¶
    :   helper for pickle

    `__reduce_ex__`()¶
    :   helper for pickle

    `__sizeof__`() → int¶
    :   size of object in memory, in bytes

    `__str__`¶

    `getForceGroup`(*self*) → int¶
    :   Get the force group this Force belongs to.

    `setForceGroup`(*self*, *group*)¶
    :   Set the force group this Force belongs to.

        |  |  |
        | --- | --- |
        | Parameters: | **group** (*int*) – the group index. Legal values are between 0 and 31 (inclusive). |

High performance molecular simulation on GPUs


### Navigation

- Application Layer
- Library Layer
  - Core Objects
  - Forces
  - Integrators
  - Extras

©2015, Stanford University and the Authors.
|
Powered by Sphinx 1.3.1
& Alabaster 0.7.7
|
Page source
